# Fragility Index of Recently Published Meta-Analyses in Pediatric Urology: A Striking Observation

**DOI:** 10.7759/cureus.16225

**Published:** 2021-07-07

**Authors:** Sachit Anand, Deepika Kainth

**Affiliations:** 1 Pediatric Surgery, Kokilaben Dhirubhai Ambani Hospital and Medical Research Institute, Mumbai, IND; 2 Pediatric Surgery, All India Institute of Medical Sciences, New Delhi, IND; 3 Pediatrics, All India Institute of Medical Sciences, New Delhi, IND

**Keywords:** fragility index, systematic review and meta-analysis, level of evidence, pediatric urology, p-value

## Abstract

Background and objective

Fragility Index (FI) of meta-analyses determines their stability in terms of the level of confidence and strength behind the results depicted by them. The present study was conducted to estimate the FI of recently published meta-analyses in the Journal of Pediatric Urology (JPUrol).

Method

Twenty recently published articles on meta-analyses were screened to identify the eligible ones. The baseline data of each meta-analysis including the details of the author, number of included studies, total sample size, the total number of events, the status of the overall outcome (significant or non-significant), type of effect measure, type of method used for pooling the estimates, and type of effects model were recorded. FI was calculated by doing each single status modification. The 95% CI of the treatment effect was re-calculated until the statistical significance of the meta-analysis was reversed.

Results

A total of seven articles incorporating 22 meta-analyses were included. Seven (32%) of them had a statistically significant outcome prior to FI estimation. The risk ratio (17/22; 77%) was the most commonly used effect measure. The random-effects model (15/22; 68%) and the Mantel-Haenszel method (20/22; 91%) of pooling the estimates were utilized in the majority of meta-analyses. The median (Q1-Q3; range) FI of statistically significant, non-significant, and total meta-analyses were 5 (3-19.5; 2-39), 5 (3.5-6; 1-17), and 5 (3-13; 1-39) respectively. FI of ≤5 was noticed in four out of seven (57%), 9/15 (60%), and 13/22 (59%) of these meta-analyses respectively.

Conclusion

Based on our findings, the majority of the recently published meta-analyses in the field of pediatric urology are fragile and depend upon the event status of ≤5 participants.

## Introduction

The first meta-analysis in medical literature was published by Karl Pearson in 1904 [1]. Since then, the number of published meta-analyses has been increasing every year. In fact, more than two lakh meta-analyses have been published on PubMed between 1966-2021. Due to this rapid increase in their number, it has become difficult to properly gauge the quality and reliability of the information disseminated by these meta-analyses [2]. The Fragility Index (FI) is one of the recently developed tools to scrutinize the strengths and robustness of clinical trials based on the highest level of evidence. FI of a meta-analysis is defined as the minimum number of changes in the event status (event to non-event and vice versa) in one or more included studies that are required to reverse the significance status (significant to non-significant and vice versa) of the meta-analyses.

FI was first devised by Walsh et al. for randomized controlled trials (RCTs) [3]. Later, Atal et al. demonstrated its application for meta-analyses of RCTs [4]. A recent study by Schröder et al. [5] has shown its wider applicability and has supported its use for meta-analyses of observational studies also. Although the application of FI for meta-analyses of non-RCTs is a point of debate, the technical challenges of conducting good-quality RCTs in surgical specialties are even greater. In the present study, an estimation of the FI for recently published meta-analyses in the Journal of Pediatric Urology (JPUrol) was performed to document their fragility. We hypothesize that the majority of the available meta-analyses in the field of pediatric urology are fragile and their outcome depends on the event status of very few participants only.

## Materials and methods

Study selection

The online archives of the JPUrol (www.jpurol.com) were searched on June 9, 2021, and 20 recently published articles with the term ‘meta-analysis’ in their title were screened. For each eligible article, all the meta-analyses reported in that paper were included. The ‘meta-analyses reporting a significant outcome’ were not considered as an explicit inclusion criterion, and those reporting a non-significant result were also included. Consistent with the previous studies [5], the inclusion of meta-analysis of studies other than RCTs was also considered. The articles examining meta-analyses of non-comparative studies were excluded. Also, those in which a pooled treatment effect was not reported were excluded.

Estimation of the Fragility Index 

The FI was calculated as done in previous studies [4,5]. After performing each event status modification, the 95% CI of the treatment effect (risk ratio or odds ratio or risk difference) was re-calculated until the statistical significance of the meta-analysis was reversed. We also confirmed our calculations of FI for each meta-analysis by comparing them with the results achieved from an online platform: http://clinicalepidemio.fr/fragility_ma/. Subsequently, the median (IQR; range) FI of statistically significant, non-significant, and all (significant + non-significant) meta-analyses were calculated and recorded.

Data extraction and analysis

The baseline data of each meta-analysis including the name of the author, year of publication, number of included studies, total sample size, the total number of events, the status of the overall outcome (significant or non-significant) prior to FI estimation, the type of effect measure used (risk ratio or odds ratio or risk difference), the type of method used for pooling the estimates (Mantel-Haenszel or inverse variance or Peto), and the type of effects model (random or fixed) were recorded in an extraction table using MS Excel (Version 15.24). The data analysis was performed using RevMan 5.4 (Cochrane Collaboration, London, UK) and Stata 12.0 (StataCorp LLC, College Station, TX).

The present study involves the collection of already available data in the archives of a journal. This primary data was used to generate secondary data. Since no direct patient contact was established during the course of this study, approval from the Institutional Review Board was not needed.

## Results

The 20 recent articles [6-25] on meta-analyses were published in the JPUrol between October 9, 2017, and May 17, 2021. Of these, only seven [19-25] were included for the calculation of FI. The remaining were excluded as they incorporated non-comparative studies [6-17] and/or none of the defined methods for pooling the estimates (Mantel-Haenszel or inverse variance or Peto) were identified in them [6-18]. Among the seven included studies, there were 22 individual meta-analyses. The characteristics of the included meta-analyses are depicted in Table 1. Only seven (32%) meta-analyses had a statistically significant outcome prior to the estimation of FI. The risk ratio (17/22; 77%) was the most common effect measure used in them. The random-effects model was used in 15 of the 22 (68%) meta-analyses, and the Mantel-Haenszel method of pooling the estimates was used in the majority (20/22; 91%) of them.

The FI of all individual meta-analyses is depicted in Table 1. The median (Q1-Q3; range) FI of statistically significant meta-analyses was 5 (3-19.5; 2-39). Four of these seven meta-analyses had a FI of 5 or less. Similarly, the median (Q1-Q3; range) FI of statistically non-significant meta-analyses was 5 (3.5-6; 1-17), with nine out of 15 having an FI of 5 or less. The combined median (Q1-Q3; range) FI of all the meta-analyses was 5 (3-13; 1-39); and of these, 13 (59%) had an absolute FI of 5 or less.

**Table 1:** Characteristics of the included meta-analyses ^#^Arranged according to the year of publication, i.e. most recent to the least; *four out of six meta-analyses in this study were eligible for the estimation of FI NS: not significant. S: significant. RR: risk ratio. OR: odds ratio. M-H: Mantel-Haenszel. IV: inverse variance. FI: Fragility Index

Author^#^	Number of studies	Sample size	Number of events	Outcome status	Effect measure	Effect model	Method of pooling	FI
Miscia et al. [24]*	5	277	19	NS	RR	Random	M-H	2
Miscia et al. [24]*	5	276	271	NS	RR	Random	M-H	4
Miscia et al. [24]*	2	53	1	NS	RR	Random	M-H	2
Miscia et al. [24]*	2	53	52	NS	RR	Random	M-H	5
Silay et al. [25]	3	167	8	S	OR	Fixed	M-H	2
Silay et al. [25]	3	167	5	NS	OR	Fixed	M-H	6
Silay et al. [25]	3	167	5	NS	OR	Fixed	M-H	6
Silay et al. [25]	3	167	16	NS	OR	Fixed	M-H	3
Silay et al. [25]	8	1055	68	NS	OR	Fixed	M-H	1
Kwenda et al. [23]	9	2564	837	NS	RR	Random	M-H	15
Kwenda et al. [23]	3	221	73	S	RR	Random	M-H	3
Anand et al. [19]	5	870	79	S	RR	Fixed	M-H	39
Anand et al. [19]	4	1201	5	NS	RR	Fixed	M-H	6
Alshafei et al. [20]	6	617	46	NS	RR	Random	M-H	4
Alshafei et al. [20]	6	617	64	S	RR	Random	M-H	5
Alshafei et al. [20]	6	617	17	NS	RR	Random	M-H	4
Alshafei et al. [20]	6	617	143	S	RR	Random	M-H	3
Chua et al. [21]	7	986	119	S	RR	Random	M-H	22
Chua et al. [21]	7	999	28	NS	RR	Random	M-H	5
Chua et al. [21]	6	615	93	S	RR	Random	M-H	17
Chua et al. [22]	20	2466	268	NS	RR	Random	IV	14
Chua et al. [22]	20	2466	231	NS	RR	Random	IV	17

## Discussion

In the evidence-based practice of medicine, meta-analyses and systematic reviews are considered to provide the highest level of evidence [26]. However, if not conducted properly, several issues can arise within them. This ultimately leads to poor quality and reliability of the information disseminated by these meta-analyses. To overcome this, compliance with the Preferred Reporting Items for Systematic Reviews and Meta-Analysis (PRISMA) guidelines [27] must be ensured. In addition, a critical appraisal of these systematic reviews and meta-analyses can be performed by using the "A MeaSurement Tool to Assess Systematic Reviews 2" (AMSTAR 2) tool [28].

Recently, the FI has been developed as an additional tool to confirm the stability of meta-analyses in terms of the confidence and strength behind the results depicted by them [4]. A meta-analysis is considered to be fragile if the status of an outcome (significant or non-significant) can be reversed by changing the event status of a few participants. In the present study, it was observed that the FI of 59% of the meta-analyses was ≤5. It means that the outcome of almost 60% of the meta-analyses depends on the event status of five or fewer participants. Upon grouping the meta-analyses on the basis of their outcome status, similar findings of low FI were observed.

Consistent results were observed by Schröder et al. [5], depicting a median (Q1-Q3; range) FI of 5 (2-11; 1-50) among the meta-analyses related to pediatric surgery. The authors have very aptly discussed the rarity of the pediatric surgical ailments as the possible reason behind the low FI among these meta-analyses. However, we would like to highlight that 14 (64%) out of the 22 meta-analyses included in the present study were regarding hypospadias, which is one of the most common congenital malformations. Also, the median (Q1-Q3; range) FI of these 14 meta-analyses was 5 (3.25-12; 1-22), with 8/14 (57%) of them having an FI of 5 or less. Therefore, the lack of stability of these meta-analyses despite a more prevalent target population underscores the importance of reporting FI among the common pediatric surgical/urological ailments, e.g., hypospadias.

On the other hand, Atal et al. [4] have demonstrated an absolute FI of 5 or less in one-third of the meta-analyses included in their study. As compared to our study, a median FI of 9 highlights better stability among the meta-analyses included by them. The possible explanation for an almost double value of median FI in their study lies in the well-defined selection criteria adopted by them. Firstly, they included only Cochrane reviews. Secondly, they excluded all meta-analyses focusing on observational studies. Finally, the number of included meta-analyses was so high (>900) that it was unlikely to be influenced by the rarity of a particular disease.

The values of FI can be influenced by a number of factors. Of substantial importance are the sample size and p-value [5]. The greater the sample size, the higher the FI (more stable). In contrast, there is an inverse relationship between FI and p-value. It is often said that FI is nothing but a surrogate representation of the p-value only [5,29]. Other factors influencing the FI include the heterogeneity of treatments across the two treatment arms, the methods used for combining study results, blinding, several types of biases or errors, etc. [4].

To the best of our knowledge, ours is the first study to focus on the estimation of FI among the meta-analyses related to pediatric urology. Unlike other studies [4], we have calculated the FI of all individual meta-analyses within an article. It has been observed that the FI not only varies among the meta-analyses of different studies but also shows a considerable variation among the individual meta-analyses of the same study [19,21,23]. Thus, for a comprehensive evaluation, the FI of all eligible meta-analyses within a study must be estimated.

The results of this study must be interpreted within the context of a few limitations. First, we have screened 20 recently published meta-analyses from one journal only. Although the reason for exploring the JPUrol was due to the fact that this journal focuses exclusively on pediatric urology, it might be possible that the FI of previously published meta-analyses from the same journal or those from other journals have a higher FI. Second, FI was initially designed for RCTs. It was later adopted for meta-analyses of RCTs as well. In the present study, we have estimated the FI of meta-analyses including studies other than RCTs as well. It is believed that the FI of meta-analyses of observational studies must also be calculated due to the obvious challenges of conducting high-quality RCTs in surgical specialties [30]. Third, the estimation of FI involves changing an event to non-event or vice versa only. The effects model (random or fixed), type of effect measure used (risk ratio or odds ratio or risk difference), and the type of method used for pooling the estimates (Mantel-Haenszel or inverse variance or Peto) are kept constant. It will be interesting to note how changes in these variables affect the FI in future studies. Finally, the FI is just a tool to provide additional statistical information about the meta-analyses. It can actually give us a good insight into the p-value. However, it does not provide any descriptive information about the results of the meta-analyses.

## Conclusions

Based on our findings, almost 60% of the recently published meta-analyses in the field of pediatric urology have high fragility and their outcomes depend upon the event status of ≤5 participants. Similar to the PRISMA guidelines, a mandatory reporting of the FI can be done to provide additional information about the p-values depicted within the meta-analyses. In fact, at the time of consideration for publication, the FI can serve as an adjunctive tool for critical appraisal of meta-analyses related to common pediatric urologic ailments.
